# Supplementary material for: Exploring the Potential Hormonal Effects of Tire Polymers (TPs) on Different Species Based on a Theoretical Computational Approach
Source: Polymers (Basel). 2023 Mar 30;15(7):1719. doi: 10.3390/polym15071719 (PMC10097371; doi:10.3390/polym15071719)
Supplement: Supplementary file 1 [file polymers-15-01719-s001.zip › polymers-2284654-supplementary.pdf]

Sumpplementary Materials

2.3. Comprehensive Biotoxicity Characterization of TMPs in Different Environmental Media—Multilayer Empowerment Method

```
import matplotlib.pyplot as plt
from sklearn.decomposition import PCA
import pandas as pd
import numpy as np
pd.set_option('display.max_rows',None)
pd.set_option('display.max_columns',None)
pd.set_option('max_colwidth',100)
```

```
data = pd.read_excel('zhuchengfen.xlsx',header = None)
```

```
data.shape
```

```
(3, 60)
```

```
x = data.iloc[:,:]
x
```

	0	1	2	3	4	5	6	7	8	9	10	11	12
0	-85.814	-134.724	-103.666	-56.265	-109.635	-120.140	-105.094	-102.452	-85.575	-60.038	-134.735	-138.866	-103.575
1	-190.042	-114.182	-157.660	-130.506	-125.605	-109.222	-84.286	-83.981	-68.699	-137.881	-103.470	-119.700	-97.682
2	-127.508	-136.188	-107.039	-49.666	-80.121	-99.354	-130.416	-129.412	-66.284	-120.806	-102.930	-100.485	-107.063

```
x.isnull().sum()
```

...

```
from sklearn.preprocessing import Normalizer
x2 = Normalizer().fit_transform(x)
x2
```

```
array([[0.2961144 , 0.25096445, 0.29933217, 0.23098963, 0.18525503,
        0.2866457 , 0.30521824, 0.26309264, 0.22085935, 0.50081894,
        0.21473371, 0.28601929],
       [0.19892452, 0.1652129 , 0.23828381, 0.40606278, 0.30575938,
        0.30333673, 0.41651118, 0.30952127, 0.37467497, 0.1972558 ,
        0.20531837, 0.1877067 ],
       [0.3088179 , 0.22399055, 0.28614915, 0.2598802 , 0.23475442,
        0.36279069, 0.41278511, 0.19535964, 0.25767816, 0.36740656,
        0.24434055, 0.22061076]])
```

```
pca_line = PCA().fit(x2)
```

```
pca_line.explained_variance_
```

```
array([3.54374966e-02, 2.55454130e-02, 8.78873169e-33])
```

```
pca_line.explained_variance_ratio_
```

```
array([5.81105376e-01, 4.18894624e-01, 1.44117946e-31])
```

```
plt.figure(figsize=[10,5])
plt.plot(np.cumsum(pca_line.explained_variance_ratio_))
plt.xticks(range(0,3))
plt.xlabel("number of components after dimension reduction")
plt.ylabel("cumulative explained variance ratio")
plt.show()
```

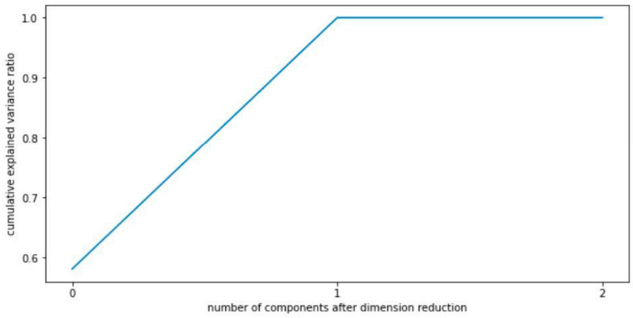


```
x3 = x2.copy()
```

```
pca = PCA(n_components =2)
pca = pca.fit(x3)
x_dr = pca.transform(x3)
```

```
x_dr
```

```
array([[-0.14419798, -0.13810033],
       [ 0.21296312, -0.03697642],
       [-0.06876514,  0.17507675]])
```

Figure S1. The code of PCA algorithm model.

2.4. Characterization of the Toxicity of SBR in Different Environmental Media—Minimum Value and Feature Aggregation Methods

```
import matplotlib.pyplot as plt
from sklearn.decomposition import PCA
import pandas as pd
import numpy as np
pd.set_option('display.max_rows',None)
pd.set_option('display.max_columns',None)
pd.set_option('max_colwidth',100)
```

```
data = pd.read_excel('DB.xlsx',header = None)
```

```
data.shape
```

```
(3, 12)
```

```
x = data.iloc[:,:]
x
```

	0	1	2	3	4	5	6	7	8	9	10	11
**0**	1.598415	1.354697	1.615784	1.246874	1.000000	1.547303	1.647557	1.420165	1.192191	2.703403	1.159125	1.543922
**1**	1.217735	1.011366	1.458676	2.485751	1.871734	1.856904	2.549712	1.894763	2.293607	1.207520	1.256875	1.149064
**2**	1.695489	1.229765	1.571032	1.426809	1.288862	1.991814	2.266296	1.072574	1.414719	2.017156	1.341492	1.211209

```
from sklearn import cluster
```

```
agglo = cluster.FeatureAgglomeration(n_clusters=1)
agglo.fit(x)
```

```
FeatureAgglomeration(n_clusters=1)
```

```
X_reduced = agglo.transform(x)
X_reduced
```

```
array([[1.50245296],
       [1.68780897],
       [1.54393474]])
```

Figure S2. The code of feature aggregation method.
